# The SH2 Domain Regulates c-Abl Kinase Activation by a Cyclin-Like Mechanism and Remodulation of the Hinge Motion

**DOI:** 10.1371/journal.pcbi.1003863

**Published:** 2014-10-09

**Authors:** Nicole Dölker, Maria W. Górna, Ludovico Sutto, Antonio S. Torralba, Giulio Superti-Furga, Francesco L. Gervasio

**Affiliations:** 1 Structural Biology and Biocomputing Programme, Spanish National Cancer Research Center (CNIO), Madrid, Spain; 2 CeMM Research Center for Molecular Medicine of the Austrian Academy of Sciences, Vienna, Austria; 3 Institute of Structural and Molecular Biology, University College London, London, United Kingdom; 4 Chemistry Department, University College London (UCL), London, United Kingdom; Fudan University, China

## Abstract

Regulation of the c-Abl (ABL1) tyrosine kinase is important because of its role in cellular signaling, and its relevance in the leukemiogenic counterpart (BCR-ABL). Both auto-inhibition and full activation of c-Abl are regulated by the interaction of the catalytic domain with the Src Homology 2 (SH2) domain. The mechanism by which this interaction enhances catalysis is not known. We combined computational simulations with mutagenesis and functional analysis to find that the SH2 domain conveys both local and global effects on the dynamics of the catalytic domain. Locally, it regulates the flexibility of the αC helix in a fashion reminiscent of cyclins in cyclin-dependent kinases, reorienting catalytically important motifs. At a more global level, SH2 binding redirects the hinge motion of the N and C lobes and changes the conformational equilibrium of the activation loop. The complex network of subtle structural shifts that link the SH2 domain with the activation loop and the active site may be partially conserved with other SH2-domain containing kinases and therefore offer additional parameters for the design of conformation-specific inhibitors.

## Author Summary

The Abl kinase is a key player in many crucial cellular processes. It is also an important anti-cancer drug target, because a mutation leading to the fusion protein Bcr-Abl is the main cause for chronic myeloid leukemia (CML). Abl inhibitors are currently the only pharmaceutical treatment for CML. There are two main difficulties associated with the development of kinase inhibitors: the high similarity between active sites of different kinases, which makes selectivity a challenge, and mutations leading to resistance, which make it mandatory to search for alternative drugs. One important factor controlling Abl is the interplay between the catalytic domain and an SH2 domain. We used computer simulations to understand how the interactions between the domains modify the dynamic of the kinase and detected both local and global effects. Based on our computer model, we suggested mutations that should alter the domain-domain interplay. Consequently, we tested the mutants experimentally and found that they support our hypothesis. We propose that our findings can be of help for the development of new classes of Abl inhibitors, which would modify the domain-domain interplay instead of interfering directly with the active site.

## Introduction

The expression of the constitutively active BCR-ABL fusion tyrosine kinase is sufficient for the initiation and maintenance of chronic myelogenous leukemia (CML) in humans [1]. BCR-ABL is the result of the t(9;22) chromosomal translocation that leads to the fusion of the Abelson tyrosine kinase (ABL1) and the breakpoint cluster region (BCR) gene [2], [3]. The dysregulated fusion protein activates a number of signaling pathways associated with inhibition of apoptosis and uncontrolled proliferation.

In the light of the above it is not surprising that the mechanisms regulating the activation and deactivation of both the wild type c-Abl and BCR-ABL tyrosine kinases have attracted a considerable interest [4]–[9].

In physiological conditions the catalytic activity of tyrosine kinases is tightly regulated through the interplay between various protein domains, phosphorylation events and associated conformational states of the catalytic domain (CD) [10]. During the catalytic cycle, its high intrinsic flexibility allows the CD to react to the regulatory elements by switching reversibly between a number of distinct active and inactive states.

**Figure 1:**
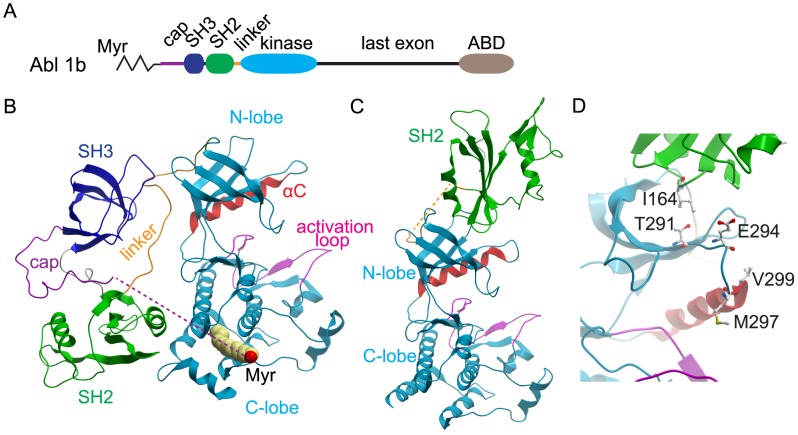
Domain organization and crystal structures of Abl kinase. **A** The c-Abl isoform Ib is characterized by myristoylation (Myr) on Gly-2 of the N-terminal capping region (cap). The tyrosine kinase domain is preceded by the SH3 and SH2 domains and a connecting linker. The last exon region contains nuclear localization signals and a C-terminal actin binding domain (ABD). **B** In the down-regulated state (PDB entry 2FO0), the SH2 domain binds the C-lobe of the kinase domain, the myristate is bound in its cognate pocket and the SH3 domain binds the SH2-CD linker. **C** In the active “top-hat” conformation (PDB entry 1OPL), the SH2 domain moves to interact with the N-lobe of the kinase domain. The αC helix and the activation loop are highlighted in red and pink, respectively. **D** Positions of the most important point mutations at the SH2-CD interface and in the β3-αC loop.

In most non receptor-type tyrosine kinases, the catalytic domain is preceded by a Src homology 2 (SH2) domain [11] (Figure 1A). The importance of the SH2 domain in the auto-inhibition and/or activation of the catalytic domain has been shown in c-Src [6], [8], [12]–[14], Hck [15]–[17], Fes [18], [19] and c-Abl, among others. The role of the SH2 domain in c-Abl is of special interest, because it is involved both in auto-inhibition and activation of the CD [18], [20], [21], and mutations in the SH2 domain have been related to imatinib-resistance in CML patients [18], [19], [22]. In the auto-inhibited state, the SH3 and SH2 domains and the SH2-kinase linker form a rigid clamp around the CD, which is locked in place by an N-terminal myristoyl modification of the N-terminal cap region inserted deeply into the CD [7], [23] (Figure 1B). This grip reduces the flexibility of the CD and, in particular, dampens the opening and closing of its N- and C-termini around the active site [16], . This so-called hinge or breathing motion of the CD is required for catalysis, and its impairment is associated with low catalytic output [26]–[28].

While the molecular basis for the role of the SH2 and SH3 domains in Abl autoinhibition is well understood, the mechanism of their activating effect is less straightforward. Recent crystal structures and small angle X-ray scattering studies have revealed that the transition from the auto-inhibited to the fully activated form of Abl requires a complete reassembly of the complex formed by the CD, the SH2 and the SH3 domains, leading to migration of the SH2 domain from the C-lobe to the N-lobe of the CD (“top-hat” conformation) (Figure 1C) [24]. Importantly, the effect of this rearrangement is not reduced to merely revoking the autoinhibition of Abl by removing the SH2-SH3 domain clamp, but the SH2 domain, when bound to the N-lobe of the CD, enhances the activity of the kinase, although it bears no direct contact to the catalytic site. Recently, it was shown that the I164E mutation in the SH2 domain, which interrupts the hydrophobic interactions at the interface between SH2 and CD in the “top-hat” conformation, leads to deactivation of Abl [5], [18]. A similar domain arrangement and activating effect has been observed in other kinases [19], [29], such as Fes [18] and Btk [30]. Hence, in multiple tyrosine kinases, the SH2 domain acts as an allosteric effector. Comparison of the crystal structures of the auto-inhibited and the activated forms of Abl does not reveal any marked conformational changes, particularly at the active site, that could explain the mechanism of activation by the SH2 domain, a finding that points towards a dynamic rather than static nature of the allosteric effect. The essential features of this allosteric effect and the molecular mechanism by which it is transferred from the N-lobe to the catalytic site still remain elusive.

The SH3/linker region has also been shown to be involved in the regulation of Abl activation [31]. However, here we focus on the SH2 domain that, even in the absence of SH3 and the linker, has been shown to have strong activating effect.

We used a multi-disciplinary approach combining elastic network models, extensive molecular dynamics simulations, free energy calculations and functional assays following mutagenesis to characterize the allosteric coupling of the CD of Abl with the SH2 domain as well as the modulation of the dynamic properties of the assembly by interactions in the “top-hat” conformation. Both the long atomistic simulations and the elastic network models indicate a significant change in the dynamics of key regulatory elements, providing a simple explanation of the mechanism of allosteric activation. Based on the computational results we designed a number of point mutants to validate the proposed model. These mutants were expressed in human cells and tested for kinase activity. We identified mutations, all distant from the active site, that were either activating the catalytic output of the kinase or were disrupting. Interestingly, we also identified a residue that when mutated lead to a decoupling of the activity of the CD from interaction with the SH2 domain. Collectively, the data suggested an effect of SH2 binding that results in changes of the properties of the αC helix, reminiscent of the effect that cyclins exert on cyclin-dependent kinases.

## Results

### The SH2 is allosterically coupled to important catalytic motifs in the kinase domain of Abl

To elucidate the mechanism by which the SH2 domain stimulates the catalytic activity of the Abl kinase we first characterized the dynamics of the CD alone and with the SH2 domain bound in the activating conformation using elastic network models. In the free CD, the two predominant modes emerging from the normal mode analysis (NMA) corresponded to the well-described hinge motion [26], [28], [32] and to a twist of N- and C-lobe against each other (Supplemental Figure S1A). When the SH2 domain was included in the elastic network model, the hinge motion continued to be the principal motion, but the corresponding normal mode included a sliding of the SH2 domain along the binding interface, while the amplitude of the movement of the N-lobe of the CD was reduced (Supplemental Figure S1B). This finding suggests that one role of the SH2 domain may be to regulate the hinge motion while restricting lobe twists during catalysis.

**Figure 2:**
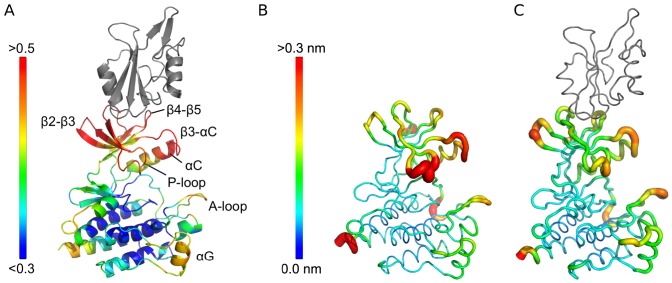
Allosteric coupling and flexibility of c-Abl. **A** Allosteric couplings of residues in the CD to the SH2 domain. High values (yellow and red) indicate strong allosteric interactions (See also Supplemental Figure S1 A–C). **B** Free CD colored by RMSF from MD simulations. **C** SH2-CD construct colored by RMSF. (See also Figure S2).

We analyzed the allosteric coupling of local conformational fluctuations along these normal modes [33] (see Methods and Text S1). Figure 2A and Supplemental Figure S1C show that local distortions in the SH2 domain are associated with conformational changes in both lobes of the CD. Important couplings are detected between the SH2 domain and specific, spatially separate motifs in the C-lobe, including the catalytically important activation loop (A-loop), the αD-αE loop, the αF-αG loop and the αG helix. The αD-αE loop forms part of the myristoyl binding pocket. The αG helix is known to have an important role in the catalytic mechanisms of many kinases. In some of them, c-Abl among them, it forms part of a platform for substrate binding [34]. Furthermore, it has been proposed that in Abl and EGFR the αF helix and the αF-αG loop are allosterically coupled to the αC helix and are involved in the dynamically enhanced stabilization of active conformations [20], [29]. Virtually all other motifs coupled to the SH2 domain in turn also couple to the crucial A-loop.

The allosteric coupling analysis thus suggests that the SH2 domain acts primarily on the N-lobe loops and the A-loop. All these motifs are coupled among them through a dense network of allosteric interactions, which affect also the P-loop and the αC helix, two structural motifs that, together with the A-loop, participate actively in the catalytic process.

### The SH2 domain modifies the dynamics of the catalytic domain

**Figure 3:**
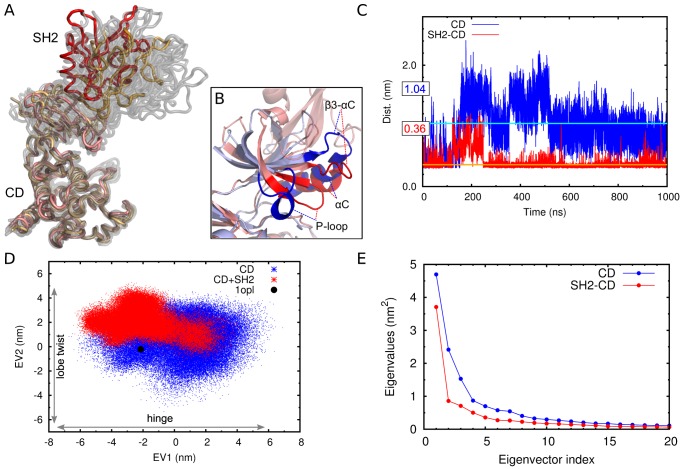
Conformational plasticity and essential dynamics of c-Abl. **A** Range of conformations explored by SH2-CD. The crystal structure is shown in red, the predominant conformation from the simulation in orange. Other representative structures from the trajectory are shown in grey. **B** Close up on the most representative structure of the CD and the CD-SH2 complex. **C** Distance between E305(Cδ) and K290(Nζ). The average values, calculated for the last 750 ns of simulation time, are marked by cyan (CD) and orange (SH2-CD) lines and labelled with the corresponding values (in nm). **D** Projection of the trajectories of the CD in absence and presence of SH2 on the two dominant eigenvectors from the principal component analysis of the trajectory of the free CD. **E** Eigenvalue spectrum for the CD and the SH2-CD constructs. (See also Figures S1 and S3).

We next carried out 1 µs long all-atom molecular dynamics (MD) simulations of the free CD and the activated SH2-CD complex in solution. The structure of the CD does not, in general, deviate much from the crystal structure (Supplemental Figure S2A, blue line). However, some motifs have a much higher degree of intrinsic flexibility than others, as can be seen from the root mean square fluctuations (RMSF) of the backbone (Figure 2B and Supplemental Figure S2B, blue lines). The largest fluctuations were observed in the N-lobe loops, the αC helix, and the A-loop. Enhanced flexibility of the N-lobe together with partial unfolding of the αC helix has been observed in other protein kinases, such as FES [18] and EGFR [35]–[37]. The flexibility of these motifs are thought to be crucial for the regulation of activity and sensitivity towards specific kinase inhibitors [38]–[40]. The partial unfolding of the αC helix results in the rupture of a conserved salt bridge (Glu305 to Lys290), which needs to be formed and maintained stable for Abl activation [41] (Figure 3C, blue line).

When the SH2 domain was included in the simulation, the CD domain is more rigid (Figure 2C and Supplemental Figure S2A, orange line), while the relative orientation of the two domains and the position of the SH2 domain change noticeably (Figure 3A and Supplemental Figure S2A, red line). Compared to the crystal structure, conformations in which the SH2 domain is shifted towards the active site predominate.

A further hint at the mechanism by which the SH2 domain is connected to the dynamics of the catalytic domain is given by the comparison of the corresponding flexibility patterns (Figure 2B, C and Supplemental Figure S2B). By far the strongest impact is on the P-loop, which is known to be vital for ATP binding and kinase selectivity [36].

Figure 3B compares the most representative structures of the CD in absence (blue) and presence (red) of the SH2 domain, obtained by a cluster analysis of snapshots from the trajectories. The SH2 domain directs the β3-αC loop towards the active site and the A-loop (Supplemental Figure S3A, B). In this conformation, the β3-αC loop is stacked over the P-loop, fixing it in a conformation in which it points towards the DFG motif in the active site and the αC helix. This interplay between β3-αC loop, P-loop and αC helix does not take place in the free CD. Furthermore, the rearrangement and stiffening of the N-lobe motifs results in a stabilization of the crucial salt bridge between Glu305 and Lys290 (Figure 3C, red lines).

While the SH2 domain strongly stabilizes the N-lobe in general and in particular the P-loop, it enhances the flexibility of the A-loop and the αF-αG loop. The A-loop plays a fundamental role in the inactive-to-active conformational switch and together with the other elements, is involved in substrate/product binding (Supplemental Figures S2C, D). It is thus conceivable that the interaction with SH2 might play a role both in the inactive-to-active equilibrium and in the release of the products, which has been proposed to be the rate determining step of c-Abl-dependent phosphorylation.

Next, we addressed the issue of how the SH2 domain affects global motions of the CD by using principal component analysis (PCA), which provides a description of the dominant motions of the CD during the simulations.

Figure 3D shows the projection of the trajectories in absence and presence of the SH2 domain on the eigenvectors (EVs) of the two most predominant modes of motion of the backbone of the CD. These principal eigenvectors match the first normal modes of the simplified elastic network model quite well, representing again the hinge motion and the lobe-twist, The only significant difference is found in the second PCA eigenvector, that also includes a distortion of the αC helix (Supplemental Figure 1D). The agreement of these two completely independent methods in describing global changes in the CD dynamics is remarkable and indicates that the conformational changes observed during the PCA are facilitated by the low-frequency, global motions that are intrinsic to the structure.

The SH2 domain significantly restricts the movement along both EVs and leads to changes in the conformational equilibrium. The amplitude of the hinge motion along EV1 is shifted towards conformations in which the lobes close down over the active site (Figure 3D, left side of the graphic, and Supplemental Figure S1D, blue lines), which is also reflected directly by the distance between N- and C-lobe (Supplemental Figure S3C). The effect of the SH2 domain on the motion along EV2 is also strong. The twist between the N- and C-lobes, which leads to conformational changes at the active site of the kinase, is constrained and the distortion of the αC helix is strongly reduced (Figure 3D). A PCA analysis of the trajectory of the SH2-CD construct revealed that, unsurprisingly, the first eigenvector still represents the pure hinge motion. However, eigenvectors 2 to 4 all include a certain degree of hinge closing but virtually no distortion of the active site motifs (Supplementary Figure S1E). The corresponding eigenvalue spectra furthermore confirm that in presence of the SH2 domain the hinge motion (EV1) gains importance relative to all other modes (Figure 3E).

The MD simulations indicate that the SH2 domain bound in the “top-hat” conformation exerts a double effect. On the one hand, it locally reduces the flexibility of a number of structural motifs of the CD, which participate in the catalytic process, such as the P-loop, the β3-αC loop and the αC helix, locking them in their active conformations. On the other hand, it modifies the collective motions of the CD, channeling energy away from unproductive twists and distortions into the catalytic hinge motion, and shifting the hinge motion towards closed conformations and the A-loop towards more “active-like” conformations.

### The SH2 domain modifies the inactive to active conformational equilibrium

To investigate the effects of the SH2 domain on the A-loop conformation propensities we computed the inactive-to-active free energy landscape. To that aim we used a multiple-replica free energy algorithm (parallel-tempering Metadynamics) and a structure based hybrid force field. A similar computational strategy has been successfully used in the case of the CD of the highly homologous Src kinase to study the A-loop opening, where it was able to provide an accurate reconstruction of the free energy surface underlying the transition [42]. This force field reproduces fairly well the flexibility patterns observed with long all-atom explicit solvent simulations, apart from a small discrepancy in the region corresponding to the αG-helix in the CD. This region appears to be somewhat more rigid that it should be in the CD, but recovers the correct flexibility in the complex (Supplemental Figure S4).

**Figure 4:**
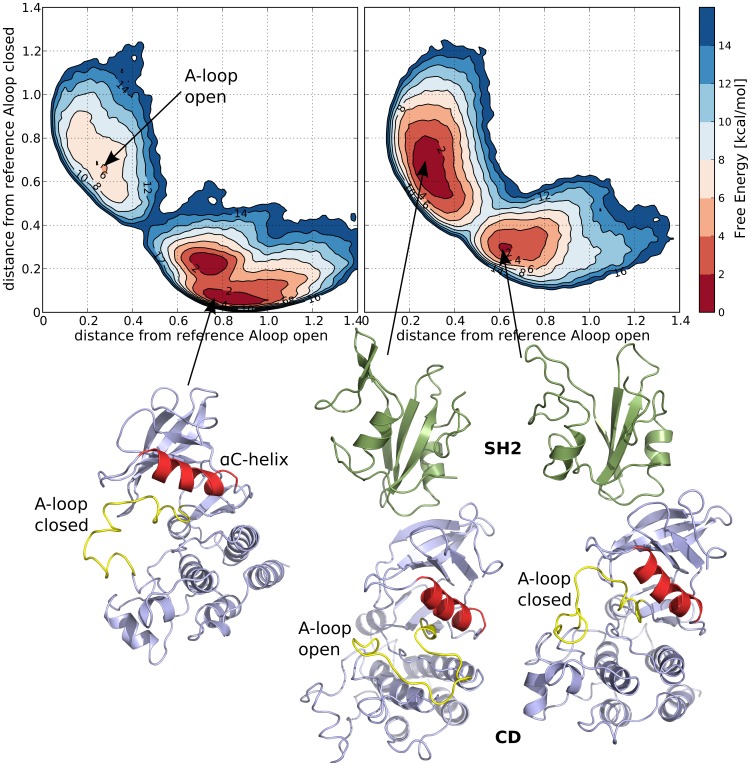
The free energy of conformational changes of the A-loop. The free energy surfaces of the A-loop transition from open (active-like) to closed (inactive-like) conformation in case of the ABL catalytic domain alone (left) and in presence of the SH2 regulatory domain in the “top-hat” conformation as a function of the contact map distances to the respective reference structures. For the deepest minima a representative structure is also shown below with the CD colored in blue, the SH2 in green, the A-loop in yellow and the aC-helix in red.

In absence of SH2, the A-loop of the CD is mostly closed, as expected from its in-vitro low catalytic activity (Figure 4, left). The A-loop active-like, or “open”, conformation is still a local minimum of the free energy but at a much higher value (ΔG_O-C_≈6 kcal/mol). Moreover, the large free energy barrier separating the closed to the open A-loop state (ΔG_‡-O_≈14 kcal/mol) disfavors the transition.

In contrast, the effect of the SH2 regulatory domain is to shift the equilibrium towards an active-like conformation of the A-loop (Figure 4, right) rendering it as stable as the closed conformation. The free energy barrier between the two states is greatly reduced (∼6 kcal/mol lower than without SH2) and the open basin is widened increasing the A-loop flexibility.

### Specific point mutations affect the CD-SH2 interplay

Based on these computational results, we proposed a number of point mutations, both in the SH2 and in the catalytic domains, that we expect to modify the dynamic interaction between the domains by interrupting essential interactions or altering the flexibility of important motifs (Table 1 and Text S1). We mainly focused on mutations in the β3-αC loop and the hinge region, as in the simulations these motifs were most strongly affected by SH2 binding. Both motifs are known to be relevant for catalysis, so mutations in these regions can be expected to affect kinase activation in general. However, based on the simulations, we predicted that for some of them the effect should be different in the free CD and in the SH2-CD construct and therefore strengthen our proposal of the how the SH2 domain interferes with c-Abl dynamics.

**Table 1:** List of c-Abl point mutants investigated in this study with summary of the effect of mutations.

Change	Region	Lysate staining		IP staining	In vitro kinase activity	Overall effect on Abl kinase activity
		a-pY	a-pY412-Abl	a-pY412-Abl		
**N165A**	SH2	++	++	++	++	++
**E187K**	SH2	+	nt	++	++	++
**K266E**	β1-β2 loop	−	nt	nt	nt	−
**E187K K266E**	SH2 + β1-β2 loop	−	nt	nt	nt	−
**T291A**	β3-αC loop	+	+	−	+	+
**T291V**		+	+	−	+	+
**T291F**		+	+	−	+	+
**T291S**		+	+	−	+	+
**M297G**	β3-αC loop	++	++	++	++/+++	++/+++(CD)
**M297L**		+	+	+	+	+
**E294P**	β3-αC loop	++	++	++	+++	+++
**V299P**		++	++	++	++	++
**E294P V299P**		++	++	+++	+++	+++
**P328G P329G**	β4-β5 loop	−	nt	nt	nt	−
**F330A**	β5	−	nt	−	−	−
**F330V**		++	++	nt	++	++
**Y339P**	hinge	+	nt	nt	−	−
**Y339G**		++	nt	nt	++	++
**G340P**	hinge	−	−	nt	nt	−

Mutants were tested for c-Abl activity via immunoblotting of HEK293 lysates or immunoprecipitates (IP) and via kinase activity assay. The numbering of residues is in agreement with the sequence of the isoform Ib. Activity scoring (effect of the mutation):, nt, not tested, − inactive (mutation disruptive), + weakly active (mutation mildly disruptive), ++ activity similar to wild-type (mutation neutral), +++ hyperactive (mutation activating) (See also Supplemental Table S1).

**Figure 5:**
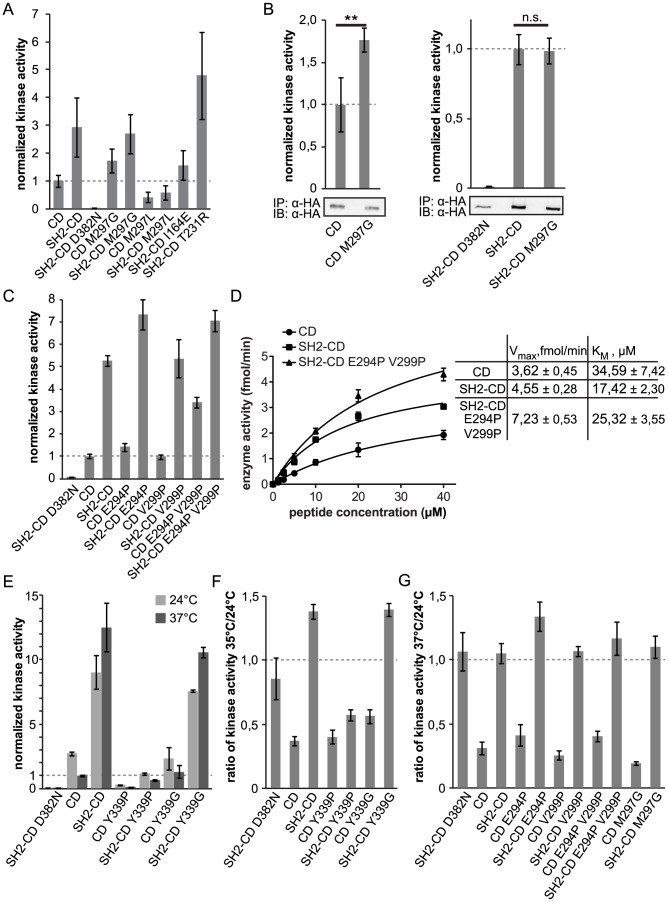
Effect of point mutations on the kinase activity of c-Abl. Abl proteins were immunoprecipitated and assayed for phosphorylation of an optimal Abl substrate peptide. The kinase activity was normalized to the amount of protein and to the activity of the wild-type CD or SH2-CD construct. **A** M297L was found to decrease kinase activity, whereas M297G had an activating effect in the absence of the SH2 domain. The kinase-dead mutant D382N presented no activity, and the known inactivating (I164E) and activating (T231R) mutations also show, accordingly, a decreased and increased activity. **B** The M297G mutant is significantly more active in the context of the isolated kinase domain, but not in the presence of the SH2 domain. The error bars are standard deviations from biological quadruplicates (n = 4, **P<0.01, Student *t* test). **C** Mutations E294P and E294P V299P have an activating effect. The reactions were performed at 37°C. **D** The activity of wild-type and E294P V299P Abl proteins was measured at increasing substrate concentrations and 25 µM ATP. **E** Y339G substitution is neutral, whereas Y339P diminishes Abl activity. The effect is seen both at 24°C and at an elevated temperature. **F, G** The ratio of the kinase activity at the elevated and room temperatures. Except for Y339P, the SH2-CD proteins retain their activity at the elevated temperature, whereas the activity of the CD constructs is reduced at least 2-fold. The error bars are standard deviations from technical triplicates except for (B). (See also Figures S4 and S5).

The effect of the mutations was characterized by assays of c-Abl kinase activity *in vivo* and *in vitro*. None of the candidate mutations have been described previously as clinically relevant and could in principle have either a moderate activating, neutral, or disruptive effect. The effect of the mutations on the catalytic activity of c-Abl was assessed both in the context of the SH2-CD module and in the isolated CD, in order to discern whether the mutation generally affects the fold or activation state of the kinase domain, or if it specifically targets the SH2-mediated regulation mechanism. The mutations were introduced in HA-tagged Abl SH2-CD or CD-only constructs which were transiently expressed in human embryonic kidney 293 (HEK293) cells, and the level of their *in vivo* activity was assessed by phospho-Y412-Abl and total phosphotyrosine immunoblotting of the crude lysates. The mutated proteins mostly accumulated to levels comparable to the wild-type constructs, indicating that they did not affect protein stability. The impact on c-Abl enzymatic activity using an *in vitro* assay with an optimal peptide substrate was assessed for selected mutations (Figure 5). As observed previously [5], under the conditions of this assay, the wild-type SH2-CD module exhibits at least 2-fold higher activity than the wt CD construct. Since the peptide substrate contains a single phosphorylation site, the observed difference in activity could be ascribed to the SH2 domain-mediated activation that is independent of phosphotyrosine binding and processive phosphorylation events that may ensue. The comparison of SH2-CD wt and SH2-CD S173N (a FLVRES motif mutant which abolishes pTyr binding by SH2) using this assay shows no difference in kinase activity, hence pTyr binding contributions can be excluded [5]. Thus, this system should be well suited to study the effects resulting from SH2-kinase domain interactions.

Some of the tested mutations were neutral, such as N165A and E187K in the SH2 domain (Supplemental Figure S5), or F330A in the β5 sheet. Others abolished kinase activity and could not be rescued by the SH2 domain, like K266E in the β1-β2 loop, P328G P329G in the β4-β5 loop, F330A in the β5 sheet, or G340P in the hinge. In these cases, the effect of the mutation goes beyond interruption of the CD-SH2 interaction and interferes directly with the catalytic mechanism.

All of the tested mutations at the T291 position in the β3-αC loop exerted a moderately disrupting effect both in the context of the SH2-CD module, as well as the CD alone, suggesting that the effect of the mutation is not likely to be explainable solely by the abolishment of the interaction with the SH2 domain (Supplemental Figure S6). It is possible that T291 plays an important role due to its localization in the key β3-αC loop, where it might be required to confer or sustain an active conformation of the kinase domain.

A number of mutants (Figure 1D), however, were more revealing in terms of understanding the SH2 activation mechanism. M297 in the β3-αC loop turned out to be very sensitive to mutations. Based on the MD simulations, we hypothesized that the β3-αC loop may act as a lever, transmitting the signal from the SH2 domain to the catalytic site and positioning the αC helix correctly. The relatively conservative M297L mutation led to a several-fold decrease in kinase activity (Figure 5A). Surprisingly, the more drastic change of the M297G mutation did not impair kinase activity, but had a slightly activating effect on the isolated CD, while it was neutral in the context of the SH2-CD construct (Figure 5A, B). The slight increase in CD activity had not been anticipated from the simulations of wt c-Abl, and suggests a *de novo* effect, which underlines the importance of this region for modulating c-Abl activity. In the presence of SH2, the activating effect of M297G was either suppressed, masked by similar changes, or compensated for by a corresponding drop in c-Abl activity. This suggests that SH2 indeed uses the β3-αC region as a key lever to efficiently redirect the conformational changes of CD. Changing the side chain and therefore the highly sensitive interaction network (M297L) severely decreases c-Abl activity, while introduction of additional flexibility (M297G) has a slightly activating effect and makes c-Abl activation less dependent on SH2 domain binding.

The pivotal role of the β3-αC loop is further confirmed by the effect of mutating E294 and V299 to prolines, which are the equivalent residues in c-Src, a protein closely related to c-Abl but not known to be activated by SH2. We would expect the E294P V299P double mutant to stiffen the β3-αC loop lever and, consequently, the αC helix. In turn, this should activate the CD and enhance the effect of the SH2 domain. The double mutant was indeed found to be markedly activating, both in the context of the catalytic domain alone as well as within the SH2-CD construct (Figure 5C). The introduction of E294P and V299P resulted in a substantial increase in enzyme velocity compared to wt SH2-CD (Figure 5D). The single mutation E294P also exerted an activating effect on Abl activity, however the effect was more pronounced in the presence of V299P, suggesting that the two mutations might act synergistically (Figure 5C).

Lastly, we have investigated the effect of changes in the flexibility of the hinge region. In the MD simulations, Y339 in the hinge region showed the highest fluctuations. Mutation of the tyrosine to glycine, which should make the hinge even more flexible, has no effect on c-Abl kinase activity (Figure 5E). In contrast, the Y339P mutation, which should rigidify the hinge region, was indeed found to be disruptive to c-Abl activity (Figure 5E). Mutation of another flexible hinge residue, G340, to proline also abrogated c-Abl activity, as observed by a decrease in phosphorylation of cellular proteins on tyrosine.

Finally, another striking evidence of the stabilizing effect of the SH2 domain on the kinase domain comes from the changes of *in vitro* measured c-Abl activity upon temperature increase (Figure 5F, G). At higher temperature, the activity of the c-Abl CD constructs decreased substantially, most likely due to an increase in mobility that is undirected and unproductive. However, under the same conditions, the activity of c-Abl SH2-CD increased, suggesting that the SH2 domain is able to direct the enhanced movements of the kinase towards more productive states, as had been indicated by the changes in the PCA eigenvalue spectrum due to SH2 binding. Interestingly, the Y339P mutation which should rigidify the hinge region, could not be rescued by an increase in temperature, and the activity of the mutant SH2-CD was only slightly raised at 35°C as compared to CD Y339P (Figure 5F). Contrary to the Y339P mutant in the hinge region, the M297G and E294P V299P mutants in the β3-αC loop preserve the effect of the temperature increase (Figure 5G), suggesting that it is, in fact, the hinge motion that is responsible for the effect. We suggest that the hinge region always has to maintain an important degree of flexibility in order for the kinase to be functional. In the free CD, conformations with a large opening of the hinge dominate, and additional twists and distortions render the hinge motion largely ineffective. Upon SH2 binding, the non-catalytic motions are strongly restricted and the hinge motion is directed to the optimal amplitude and opening needed for catalysis.

### Simulations of selected mutants reveal the origin of their effect

In the experiments, the M297G single mutant and the E294P V299P double mutant modified the activity of the CD as well as the effect of the SH2 domain in the activating “top-hat” conformation. Based on the simulations of the wt kinase, we had predicted that changes in the flexibility of the β3-αC loop should affect the directing effect the SH2 domain exerts on the CD. We carried out unbiased 1 µs simulations of the free CD and the CD-SH2 construct of both mutants to gain insight at atomic level into the mechanism underlying the observed effects and test our hypothesis.

The M297G mutant, which was chosen to introduce additional flexibility in the β3-αC loop and weaken the coupling of the SH2 domain with the αC helix, was slightly activating in the free CD and neutral in the CD-SH2 construct. In the simulation of the mutant CD, the expected enhanced flexibility of the β3-αC loop was confirmed (Supplemental Figure S7A). The simulation also provided us with an explanation for the rather unexpected increase in activity of the CD. The expansion of accessible conformations lead to the loss of interactions between the β3-αC and the P-loop, and enabled the system to assume a conformation similar to that observed in the wt in the presence of SH2 without requireing the directing effect of the SH2 domain (Supplemental Figure S8A, C). Contrary to the wild type, the flexibility of the CD in the M297G complex was enhanced, not reduced upon SH2 binding (Supplemental Figures S7B, S8B), and no additional strengthening of the salt bridge between the αC helix and the β3 sheet was observed (Supplemental Figure S7C). The picture emerging from the simulations is thus that the M297G mutant in the CD partly mimics SH2 binding. At the same time, due to the enhanced flexibility of the N-lobe loops, the effect SH2 domain on the arrangement of the catalytic important motifs is much smaller than in the wt, leading to a situation, where the difference in activity between CD and SH2-CD is markedly reduced.

To summarize, the simulations indicate that introduction of a glycine residue in the β3-αC loop enhances the flexibility of the N-lobe, allowing the P-loop to adopt an activated conformation, while it partly uncouples kinase activation from SH2 domain binding.

In the E294P V299P double mutant, the introduction of two proline residues in the β3-αC loop was expected to stiffen it, limiting the distortions of the αC helix. In our experiments, the E294P V299P double mutant was, indeed, found to be significantly activating, while, in contrast to the M297G mutant, the enhancing effect of the SH2 domain was preserved.

In the MD simulation, the mutant CD exhibited a flexibility pattern very similar to the wt with a general reduction in flexibility, which was strongest in the P-loop, the β3-αC loop and the αC helix (Supplemental Figure S7D and Supplemental Figure S8D). Surprisingly, the SH2 domain, however, increased the conformational fluctuations (Supplemental Figure S7E). Closer inspection of the corresponding structures, however, showed that this is due to concerted movements of P-loop, β3-αC loop and αC helix, which follow the movement of the SH2 domain and enhance the hinge motion (Supplemental Figure S8E). The essential salt bridge between E305 and K290 is strongly stabilized even in absence of SH2 (Supplemental Figure S7F). In principle, the substitution of Glu294, in close vicinity of the SH2 domain, could alter the domain-domain interplay, above and beyond changes in flexibility, due to the loss of possible electrostatic interactions between the glutamate side chain and residues of the SH2 domain. We compared the essential dynamics of wt and mutant, and found that in the free CD the hinge movement and the lobe twist are strongly affected by the mutation, while they remain practically unchanged in SH2-CD (Supplemental Figure S8F, G). This finding supports the view that it is the altered dynamical properties rather than lost interactions between E294 and the SH2 domain that leads to the changes in kinase activation.

## Discussion

The combination of structure-based normal mode analysis, unbiased MD simulations and free energy calculations with experiments *in vitro* and *in vivo* has shown how the SH2 domain in the “top-hat” conformation changes the dynamics of the catalytic domain of the Abl kinase. Based on the MD simulations, we could identify three residues (M297, E294 and V299) that are key to the interplay between catalytic and SH2 domain in different ways.

Contrary to what could have been expected, the residues forming the hydrophobic spine are not directly affected by the formation of the activating complex. The picture that emerges from our studies is that the SH2 domain stabilizes and repositions the loops that form the binding site, which, in turn, interact with the surrounding loops, stabilizing the P-loop and the αC helix, redirecting the movement of the N-lobe and changing the inactive to active conformational equilibrium of the A-loop. The stabilization and repositioning of the αC helix also leads to an enhancement of the salt bridge between E305 and K290, which has been identified as one of the structural elements essential for kinase activation. The β3-αC loop preceding the αC helix is the key player in this complex mechanism, acting as a lever that transmits the effect from the SH2 domain-binding site towards the substrate binding residues and the catalytically important motifs. The effects of the M297G and E294P V299P mutants demonstrate the crucial role of the β3-αC loop. The observation that these mutations have a different impact on the CD and on the SH2-CD constructs shows that the β3-αC loop is involved in Abl activation by the SH2 domain. While the introduction of a glycine breaks the chain of transmission from the SH2 domain towards the active site and uncouples SH2 domain binding and kinase activation, the stiffening of the β3-αC lever by the introduction of two proline residues enhances the activating effect of the SH2 domain.

The finding that at higher temperatures the unbound catalytic domain becomes less effective while the activity of the CD-SH2 construct increases indicates that the main role of the SH2 domain is to channel kinetic energy into directed, catalytically relevant motions. We propose that the SH2 domain modifies the so-called hinge motion, an opening and closing of the N-and C-lobes upon the active site. This is underlined by the fact that stiffening the hinge by the Y339P mutation decreases substantially the activity of the free CD, which cannot be rescued in the CD-SH2 construct. The hinge motion and the other changes in dynamics upon SH2 domain binding are involved in both the inactive-to-active conformational changes of the A-loop and the release of the products, the phosphorylated substrate and ADP. The latter has been proposed to be the rate-determining step of the phospho-transfer reaction in some kinases [38], [43]. Analysis of the flexibility pattern of the M297G and E294P V299P mutants reveals that the SH2 domain does not simply stabilize catalytically important motifs but that it regulates the Abl kinase through a complex interplay between stabilization, subtle local conformational changes and direction of the concerted hinge motion.

It is interesting to note that the effect on the β3-αC loop are reminiscent of the binding of cyclin to cyclin-dependent kinases [44], [45] and the dimerization of the EGF Receptor, in which the catalytic domain of the activator kinase engages the helix αC of the receiver kinase [46]. The effect of the SH2 domain in Abl, however, occurs at a distance, exploiting a network of interactions and motions that effectively may be equivalent to interaction with the so-called αC “hydrophobic patch” by different structural elements in other kinases, such as the mentioned CDKs, EGFR but also Aurora, Rho kinase and other (reviewed in [41]). Interestingly, Fes, the prototypic SH domain containing tyrosine kinase, engages the SH2 domain also in a “top hat” position relative to the kinase domain, but here the SH2 domain reaches down sufficiently to interact directly with the αC helix [18], [19]. The discovery of the allosteric coupling of catalytically active residues in Abl with SH2 binding may instruct strategies aiming at the development of small molecules interfering with the SH2-kinase interface, but also conformation-depending kinase inhibitors [5], [47], [48]

## Methods

### Allosteric coupling analysis

Normal mode analyses (NMA) were applied to elastic network models of the CD alone and with the SH2 domain bound at the N-lobe in the activating conformation. The models were built from the crystal structure of the CD-SH2 complex (pdb-id: 1opl, chain B).

Allosteric couplings were calculated following the approach of Ref. [33]. They measure correlations in structural distortions along protein normal modes, quantifying interactions between distant residues. See also Text S1.

### Molecular dynamics simulations

The CD of c-Abl with the SH2 domain bound in the top-hat conformation (PDBid: 1OPL, chain B) was solvated in tip3p water molecules [49], and the system charges were neutralized, adding the proper number of positive (Na+) or negative (Cl−) ions. For the free CD, the SH2 domain was removed from crystal structure and the protein prepared accordingly. Simulations were performed with the AMBER99SB**-ILDN force field [50], using GROMACS 4.5 [51].

The free energy calculations were performed with parallel tempering metadynamics (PT-metaD) [52], and a structure based hybrid force field.

For details on the set-up of the simulations and the coarse-grained model see Text S1.

Root mean square deviations (RMSD), root mean square fluctuations (RMSF), clustering, distances and principal component analysis were performed with GROMACS tools (g_rms, g_rmsf, g_cluster, g_dist, g_covar and g_anaeig).

All structural figures were created using PyMol [53], except Figure 1 where ICM Browser was used (Molsoft L.L.C), and Supplemental Figures S1A, B where VMD was used [54].

### DNA constructs

Point mutations were obtained using the Quikchange Site-directed Mutagenesis kit (Stratagene) and pcDNA3.1-2×HA-TEV c-Abl CD and pcDNA3.1-2×HA-TEV c-Abl SH2-CD as templates [5].

### Transfection, immunoprecipitation and immunoblotting

Transfection of HEK 293 cells and immunoprecipitation of HA-tagged Abl protein was carried out as described previously [5], [7] using the monoclonal Anti-HA agarose conjugate (Clone HA-7, Sigma-Aldrich). Immunoblotting was done using the following antibodies: anti-phosphotyrosine (4G10, Millipore), anti-phospho-Abl (Tyr412, Cell Signaling Technology), and anti-HA labeled with AlexaFluor 680 (Molecular Probes). Secondary antibody was goat anti-mouse IgG labeled with AlexaFluor 680 (Molecular Probes). Alternatively, peroxidase-labeled mouse anti-HA antibody (HA-7, Sigma) was used. Quantification of the relative amounts of immunoprecipitated Abl protein was done using the Odyssey system (Li-Cor) or ImageJ program.

### Kinase assay

The in vitro kinase assays were carried out as described previously [5], [7]. Immunoprecipitated HA-tagged c-Abl constructs were resuspended in kinase assay buffer (20 mM Tris-Cl pH 7, 10 mM MgCl2, 1 mM DTT). A peptide with the preferred c-Abl substrate sequence carrying an N-terminal biotin (biotin-GGEAIYAAPFKK-amide) was used as substrate. The reaction was done at 24°C unless otherwise indicated. The terminated reaction was spotted onto a SAM2 Biotin Capture membrane (Promega). The activity was normalized for the relative amount of immunoprecipitated protein and for the activity of c-Abl CD wild-type. For the measurements of K_M_, each experiment was normalized for a reaction performed in the absence of the peptide, and the amount of kinase.

## Supporting Information

Figure S1 **Global dynamics and allosteric coupling.** (A, B) Schematic representation of the first and second normal modes of the elastic network model of the free CD (A) and the SH2-CD construct (B). Coloring by B-factors along the corresponding normal modes (from blue, lowest, to red, highest). Red arrows represent the direction and amplitude of motion of the individual residues along the normal modes. (C) Allosteric couplings in the SH2-CD complex. (D) Schematic representation of the first two eigenvectors from the principal component analysis of the MD simulation of the free CD. (E) The first two eigenvectors from the PCA of the MD simulation of the SH2-CD construct.
(EPS)

Figure S2 **Flexibility of wild-type Abl.** (A) Backbone root means square deviation (RMSD) of free CD (blue) and CD-SH2 complex (red), aligned on all backbone atoms. In orange, RMSD of the CD within the CD-SH2 complex, aligned on the backbone atoms of the CD. (B) Backbone root mean square fluctuations (RMSF) of the CD, unbound (blue) and in the CD-SH2 complex (red). For the analysis, only the last 750 ns of simulation were taken into account, in order to ensure full previous equilibration of the structures. Important structural motifs are marked in grey. (C,D) Abl in complex with an ATP-peptide conjugate (PDBid: 2G1T). Structures are colored by the RMSF from the MD simulations of the free CD (C) and the CD-SH2 complex (D). ATP is shown in a stick representation in grey and the pseudo-substrate in black.
(EPS)

Figure S3 **Conformational plasticity and hinge motion of wt c-Abl.** (A,B) Most representative structures of the CD in absence (A) and presence (B) of the SH2 domain. The representative structure of the largest cluster is shown in green and the one corresponding to the smallest cluster in blue. Upper panel: structures aligned on the C-lobe of the CD. Lower panel: detail of the αC helix, with the structures aligned on the N-lobe. (C) Distance between the Cα atoms of V299, at the N-terminus of the αC helix, and Y530, at the C-terminus of the activation loop during the simulations as a measure of the hinge motion. Top: Visualization of the corresponding residues in the CD. Bottom: distance in the free CD (in blue), and the SH2-CD (in red). The corresponding averages and standard variations are marked in grey, and the corresponding values are shown.
(EPS)

Figure S4 **Flexibility of the all-atom versus the coarse-grained models.** The average residue root mean square fluctuation (RMSF) for the CD in the open A-loop conformation is shown in the case of 100 ns of free molecular dynamics with and without SH2 in the top-hat position (solid red and blue lines respectively). The same quantities are shown with dashed lines in the case of the coarse-grained model.
(TIF)

Figure S5 **Effect of mutations of residues in the SH2 domain on c-Abl activity.** HEK293 cells were transfected with wild-type or mutant c-Abl constructs, and the lysates and immunoprecipitated proteins were analyzed by immunoblotting. E187K and N165A mutations are slightly disruptive to c-Abl activity as judged by total phos- photyrosine content in cell lysates (A). The same effect is seen when constructs were immunoprecipitated and subjected to in vitro kinase assays in the presence of an opti- mal Abl substrate peptide containing a single phosphorylation site (B). Activity of the mutants was normalized to SH2-CD wt. Error bars represent standard deviations from technical triplicates.
(EPS)

Figure S6 **Effect of mutations of residue T291 on c-Abl activity.** HEK293 cells were transfected with wild-type or mutant c-Abl constructs, and the lysates and immunoprecipitated proteins were analyzed by immunoblotting. Mutations in T291 are disruptive to c-Abl activity as judged by total phosphotyrosine content in cell lysates (A), the activity of the immunoprecipitated mutants in in vitro assays and their phos- phorylation on Y412 (B). Kinase activity was normalized to CD wt. Error bars represent standard deviations from technical triplicates.
(EPS)

Figure S7 **Flexibility and structures of the M297G mutant and the E294P V299P double mutant from MD simulations.** (A–C) Structures of CD (A) and SH2-CD (B) of the M297G mutant, colored by RMSF, and distance between E305(Cδ) and K290(Nζ) (C) in CD (blue lines) and SH2-CD (red lines). (D–F) Structures of CD (D) and SH2-CD (E) of the E294P V299P double mutant, colored by RMSF, and distance between E305(Cδ) and K290(Nζ) (F) in CD (blue lines) and SH2-CD (red lines). Average values of the E305-K290 distance, calculated for the last 750 ns of simulation time, are marked by cyan (CD) and orange (SH2-CD) lines and labelled with the corresponding values (in nm).
(EPS)

Figure S8 **Conformational plasticity and flexibility of the M297G and E294P V299P mutants.** (A–C) Conformation of the M297G CD. Most representative structures of the CD in absence (A) and presence (B) of the SH2 domain. The representative structure of the largest cluster is shown in green and the one corresponding to the small- est cluster in blue. Upper panel: structures aligned on the C-lobe of the CD. Lower panel: detail of the αC helix, with the structures aligned on the N-lobe. (C) Comparison of the P-loop and the β3-αC loop in the wt CD (upper panel, in blue), the wt CD-SH2 (middle panel in red) and the M297G CD (lower panel in cyan). (D, E) Conformation of the E294P V299P mutant. Most representative structures of the CD in absence (D) and presence (E) of the SH2 domain. (F, G) Essential dynamics of the E294P V299P mutant compared to the wt. Projection of the trajectories of the wild type and E294P V299P mutant on eigenvectors 1 (hinge motion) and 2 (lobe twist) of the principal component analysis of the trajectory of the wt. (F) CD wt (blue dots) vs. CD E294P V299P (cyan dots). (G) SH2-CD wt (red dots) vs. SH2-CD E294P V299P (orange dots).
(EPS)

Text S1 **Supplemental Methods. Table S1.** Mutants tested for an effect on the activation of the CD and SH2-CD constructs.
(PDF)
