# Supplementary figures and images for: The SH2 Domain Regulates c-Abl Kinase Activation by a Cyclin-Like Mechanism and Remodulation of the Hinge Motion
Source: PLoS Comput Biol. 2014 Oct 9;10(10):e1003863. doi: 10.1371/journal.pcbi.1003863 (PMC4191882; doi:10.1371/journal.pcbi.1003863)

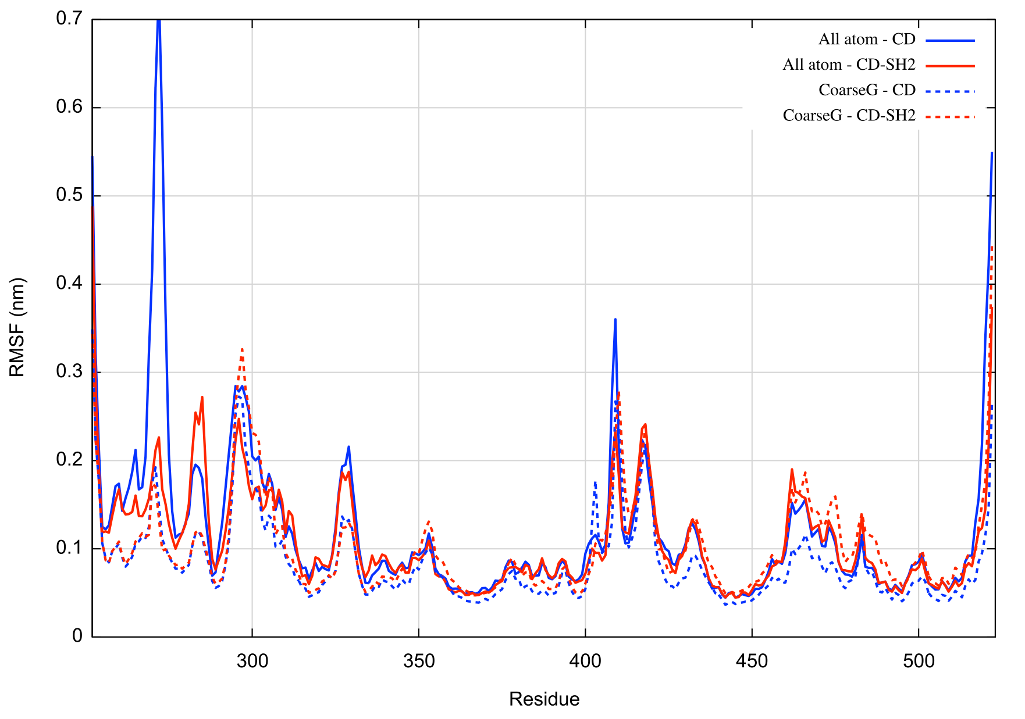

Supplement: Figure S4 — Flexibility of the all-atom versus the coarse-grained models. The average residue root mean square fluctuation (RMSF) for the CD in the open A-loop conformation is shown in the case of 100 ns of free molecular dynamics with and without SH2 in the top-hat position (solid red and blue lines respectively). The same quantities are shown with dashed lines in the case of the coarse-grained model. (TIF) [file pcbi.1003863.s004.tif]
