# Supplementary material for: The SH2 Domain Regulates c-Abl Kinase Activation by a Cyclin-Like Mechanism and Remodulation of the Hinge Motion
Source: PLoS Comput Biol. 2014 Oct 9;10(10):e1003863. doi: 10.1371/journal.pcbi.1003863 (PMC4191882; doi:10.1371/journal.pcbi.1003863)
Supplement: Text S1 — Supplemental Methods. Table S1. Mutants tested for an effect on the activation of the CD and SH2-CD constructs. (PDF) [file pcbi.1003863.s009.pdf]

Supporting Text S1

The SH2 domain regulates c-Abl kinase activation by a cyclin-like mechanism and redirection of the hinge motion

Nicole Dölker, Maria W. Górna, Ludovico Sutto,
Antonio S. Torralba, Giulio Superti-Furga,
and Francesco L. Gervasio

Supplemental Methods

Allosteric Coupling Analysis

Allosteric couplings were calculated following the coarse-grained method of Balabin, Yang and Beratan [1, 2]. Allosteric couplings measure correlations in structural distortions along protein normal modes, and therefore they quantify allosteric interactions between potentially distant residues. Similarly to mean square fluctuations, contributions of all modes are included, but unlike the standard definition [3], the effect of translations and rotations is removed, thus improving the contrast between coupled and uncoupled regions, as compared to the usual covariance maps. Briefly, the effect of fluctuations on local structural perturbations was assessed by imposing a cutoff around each coarse-grained atom, then moving the atoms within the enclosed region along the unitary normal modes obtained from an Anisotropic Network Model (ANM) [1, 3]. Rotations and translations were removed by aligning the shifted atoms back to the original structure [2], so that the mean square deviation of the alignment quantifies only structural distortions. A measure of allosteric coupling then results from crossing mean square deviations of different, potentially distant regions, along a given normal mode. Furthermore, each coefficient is a sum over all normal modes, thus incorporating the global dynamics of the protein. We chose Cα atoms to be the coarse-grained atoms and set the cut-off around each atom to 9–11 A, a range which has been observed to be large enough to include a sufficient number of neighbors while still resolving individual amino acids. Occasionally, small variations of the cutoff were necessary to avoid singular alignment matrices, which occur when there are not enough atoms within a cutoff radius. We used a slightly modified version of the ANM, after a suggestion by Balabin et al. [1]. Whereas in the standard model, spring constants between coarse-grained atoms show a stepwise dependence on the distance, it was found that a smooth sigmoidal dependence is more robust to small changes in the protein conformation.

Molecular Dynamics Simulations

The systems were enclosed in a dodecahedral box, with periodic boundary conditions and a minimum distance of 1.0 nm between the protein and the boundaries. All simulations were performed in the NpT ensemble. The temperature was kept constant using the velocity rescaling method [4] at T = 300 K. The pressure was coupled to a Parrinello-Rahman barostat [5]. All bonds were constrained [6]. An integration time step of 2 fs was used. Lennard-Jones and direct electrostatic interactions were calculated with a cutoff of 1.0 nm; long-range electrostatic interactions were calculated by particle-mesh Ewald summation [7]. The AMBER99SB**-ILDN force field [8], used for all simulations, includes the dihedral corrections of Best and Hummer [9] and the Amber03 charges on charged residues [10].

The structural clustering was done with the gromos clustering method [11] with an RMSD cutoff 0.2 nm. The PCA were performed from 150 ns to 500 ns of the simulations.

Free Energy Calculations

The hybrid coarse-grained model used to simulate the effects of SH2 on the A-loop dynamics is fully described in Ref. [12]. Briefly, it consists of an all-heavy atoms, multiple basins, structure-based model where the bonded interactions and 1-4 short-range van der Waals potentials are taken from the AMBER99SB force-field while the non-bonded interactions are given by a structure-based potential acting on a set of native contacts. This contact set comprises all the unique pairs of atoms belonging to non-neighbour residues that are within 5 Å of each other with no other atoms in-between in the vacuum energy-minimized structures of either the CD with open A-loop (PDB 2FO0), the CD with closed A-loop (PDB 2G1T) or the SH2-CD complex (PDB 1OPL).

The structure based potential is implemented as a Lennard-Jones potential whose r_0 correspond to the native distance and whose depth ϵ_0 is set to 5.0 kJ/mol for the native contacts of the closed A-loop structure and to 3.8 kJ/mol for the remaining. The value 3.8 has been previously tuned for this model to correctly reproduce a protein thermodynamics and fluctuations [12], while the enhanced stability of the closed A-loop is reflected by an increased weight of its native contacts energy. All the parameters and the set of native contacts for the catalytic domain are identical for both simulations (CD alone, CD in complex with SH2).

The sampling is performed through Langevin dynamics with the molecular dynamics package GROMACS 4 [13], with an inverse friction constant of 1 ps and an integration time step of 4 fs. To enhance the sampling and guarantee a converged free energy surface we applied the PT-MetaD technique [14] where 8 replicas of the system are run for 200 ns at increasing temperatures (T= 300, 313, 326, 340, 354, 369, 384, 400 K) and an exchange is attempted with a Metropolis rule every 40 ps. Each replica is then sampled using the metadynamics algorithm on two collective variables (CVs): the distance in the contact map space to a reference A-loop open conformation (CV1) and the distance in the contact map space to a reference A-loop closed conformation (CV2). A hill of initial height 15 kJ/mol and width 1.0 is deposited every 4 ps and decreased according to the well-tempered algorithm with a bias-factor of 5. The metadynamics calculations where carried out with the PLUMED plugin for GROMACS [15].

References

[1] Balabin IA, Yang W, Beratan DN (2009) Coarse-grained modeling of allosteric regulation in protein receptors. Proc Natl Acad Sci USA 106: 14253–14258.

[2] Kabsch W (1976) A solution for the best rotation to relate two sets of vectors. Acta Cryst A 32: 922–923.

[3] Atilgan AR, Durell SR, Jernigan RL, Demirel MC, Keskin O, et al. (2001) Anisotropy of fluctuation dynamics of proteins with an elastic network model. Biophys J 80: 505–515.

[4] Bussi G, Donadio D, Parrinello M (2007) Canonical sampling through velocity rescaling. J Chem Phys 126: 014101.

[5] Parrinello M, Rahman A (1981) Polymorphic transitions in single crystals: A new molecular dynamics method. J Appl Phys 52: 7182–7190.

[6] Hess B, Bekker H, Berendsen H, Fraaije J (1997) LINCS: A linear constraint solver for molecular simulations. J Comput Chem 18: 1463–1472.

[7] Essmann U, Perera L, Berkowitz ML, Darden T, Lee H, et al. (1995) A Smooth Particle Mesh Ewald Method. J Chem Phys 103: 8577–8593.

[8] Lindorff-Larsen K, Piana S, Palmo K, Maragakis P, Klepeis JL, et al. (2010) Improved side-chain torsion potentials for the Amber ff99SB protein force field. Proteins 78: 1950–1958.

[9] Best RB, Hummer G (2009) Optimized Molecular Dynamics Force Fields Applied to the Helix-Coil Transition of Polypeptides. J Phys Chem B 113: 9004–9015.

[10] Piana S, Lindorff-Larsen K, Shaw DE (2011) How robust are protein folding simulations with respect to force field parameterization? Biophys J 100: L47–9.

[11] Daura X, Gademann K, Jaun B, Seebach D, van Gunsteren WF, et al. (1999) Peptide Folding: When Simulation Meets Experiment. Angew Chem Int Ed Engl 38: 236–240.

[12] Sutto L, Mereu I, Gervasio FL (2011) A Hybrid All-Atom Structure-Based Model for Protein Folding and Large Scale Conformational Transitions. J Chem Theory Comput 7: 4208–4217.

[13] Hess B, Kutzner C, Van Der Spoel D, Lindahl E (2008) GROMACS 4: Algorithms for Highly Efficient, Load-Balanced, and Scalable Molecular Simulation. J Chem Theory Comput 4: 435–447.

[14] Bussi G, Gervasio FL, Laio A, Parrinello M (2006) Free-energy landscape for beta hairpin folding from combined parallel tempering and metadynamics. J Am Chem Soc 128: 13435–13441.

[15] Bonomi M, Branduardi D, Bussi G, Camilloni C, Provasi D, et al. (2009) PLUMED: A portable plugin for free-energy calculations with molecular dynamics. Comp Phys Commun 180: 1961–1972.

Supplemental Tables

Change	Region	Rationale
N165A	SH2	Interacts with β1-β2 and β3-αC loops.
E187K	SH2	Should interrupt salt bridge between E187 and K266 observed in the simulations.
K266E	β1-β2	Should interrupt salt bridge between E187 and K266.
E187K K266E	SH2, β1-β2	Should recover salt bridge between SH2 and CD.
T291A<br>T291V<br>T291F<br>T291S	β3-αC	Forms a hydrogen bond to the SH2 domain during the simulation.<br>V is conservative in the steric effects, disruptive of the H-bond.<br>F is more bulky, also disruptive of the H-bond.<br>S is expected to be almost neutral (confirm the importance of the H-bond).
M297G<br>M297L	β3-αC	Uncouple the movement of the loop from that of αC.<br>L should be almost neutral.
E294P	β3-αC	Stiffen the loop.
V299P	β3-αC	Stiffen the loop.
E294P V299P	β3-αC	Stiffen the loop.
P328G P329G	β4-β5	Uncouple movement of SH2 from β4, β5 and, consequently, αC.
F330A<br>F330V	β5	Bulkiest residue in the hydrophobic cluster between β4, β5 and αC. Disturb the transmission of the SH2-induced movement of the β-sheets to αC.<br>V is less drastic then A.
Y339P<br>Y339G	hinge	Residue in the hinge, which shows the highest flexibility. Stiffen the hinge to reduce the movement along the first mode.<br>Contrary approach, make hinge more flexible.
G340P	hinge	Stiffen the hinge.

Table S1, Related to Table I: Mutants tested for an effect on the activation of the CD and SH2-CD constructs.
